# Cycle Tracks and Parking Environments in China: Learning from College Students at Peking University

**DOI:** 10.3390/ijerph14080930

**Published:** 2017-08-18

**Authors:** Changzheng Yuan, Yangbo Sun, Jun Lv, Anne C. Lusk

**Affiliations:** 1 Department of Nutrition, Harvard T.H. Chan School of Public Health, Boston, MA 02115, USA; chy478@mail.harvard.edu; 2 Department of Epidemiology, Harvard T.H. Chan School of Public Health, Boston, MA 02115, USA; 3 Department of Epidemiology & Biostatistics, Peking University School of Public Health, Beijing 100191, China; yangbo-sun@uiowa.edu; 4 Department of Epidemiology, College of Public Health, University of Iowa, Iowa City, IA 52242, USA

**Keywords:** perceived safety, bicycle route, cycle track, bicycle parking

## Abstract

China has a historic system of wide cycle tracks, many of which are now encroached by cars, buses and bus stops. Even with these conditions, college students still bicycle. On campuses, students park their bikes on facilities ranging from kick-stand-plazas to caged sheds with racks, pumps and an attendant. In other countries, including Canada, some of the newer cycle tracks need to be wider to accommodate an increasing number of bicyclists. Other countries will also need to improve their bike parking, which includes garage-basement cages and two-tiered racks. China could provide lessons about cycle tracks and bike parking. This study applied the Maslow Transportation Level of Service (LOS) theory, i.e., for cycle tracks and bike parking, only after the basic needs of safety and security are met for both vehicle occupants and bicyclists can the higher needs of convenience and comfort be met. With random clustering, a self-administered questionnaire was collected from 410 students in six dormitory buildings at Peking University in Beijing and an environmental scan of bicycle parking conducted in school/office and living areas. Cycle tracks (1 = very safe/5 = very unsafe) shared with moving cars were most unsafe (mean = 4.6), followed by sharing with parked cars (4.1) or bus stop users (4.1) (*p* < 0.001). Close to half thought campus bike parking lacked order. The most suggested parking facilities were sheds, security (guard or camera), bicycle racks and bicycle parking services (pumps, etc.). If parking were improved, three quarters indicated they would bicycle more. While caged sheds were preferred, in living areas with 1597 parked bikes, caged sheds were only 74.4% occupied. For the future of China’s wide cycle tracks, perhaps a fence-separated bus lane beside a cycle track might be considered or, with China’s recent increase in bike riding, shared bikes and E-bikes, perhaps cars/buses could be banned from the wide cycle tracks. In other countries, a widened cycle track entrance should deter cars. Everywhere, bike parking sheds could be built and redesigned with painted lines to offer more space and order, similar to car parking.

## 1. Introduction 

Bicycling [1,2,3], a physical activity that can be a daily form of transportation, has been identified as an effective way to address sedentary lifestyle and obesity [4,5,6]. Bicycling could also produce environmental health benefits including reduced pollution, carbon emission and traffic congestion [7]. The key is getting more people to bicycle.

To foster bicycling, bicycle environments preferred by the most people should be provided [2,3,8,9,10,11,12]. Much continues to be learned about bicycling routes from the Netherlands, Denmark, Canada and the U.S. [13], but new lessons could come from the bicycle kingdom, China, where routes, bicycles and parking differ from that of other countries [14]. The primary bicycle travel route in China includes cycle tracks or barrier-protected bicycle-exclusive paths beside sidewalks, which have been suggested as safer compared to bicycling in the road [12,15]. In China, these cycle tracks have been 22 feet-wide with 18 foot-wide parallel landscaped islands [14,16,17,18,19]. During the time of the People’s Republic of China, these wide cycle tracks were for bicyclists only and served as bicycle-mass transit routes to and from work. Wide cycle tracks allowed for more bicyclists, side-by-side riding, passing and three-wheeled cargo bikes [18,20]. Since then, car use in China has increased, and cycling is now not as safe or delightful [16,21], yet many individuals, especially college students, still bicycle. In 2007 in Beijing, there were approximately 13 million bicycles, and about six million individuals bicycled every day across the city, which accounts for more than 30% of the total traffic volume [16]. The wide cycle tracks still accommodate bicyclists, but now they also have moving cars, parked cars and bus stops, which greatly reduce the speed, safety and operational flow of bicyclists. 

In addition to having a comfortable bicycle route, bicycle parking has been identified as a critical component [22,23,24,25,26,27,28]. The typical bicycle in China includes a kickstand and built-in lock, as found in the Netherlands, but not in the U.S. For college students, this bicycle is appropriate because the customary bicycle parking on Chinese campuses includes wide paved plaza areas where students can randomly park and lock their kickstand-outfitted bicycles. Even though parking in this location is considered secure, Chinese campuses also provide caged sheds which, in some locations, have staff to repair bicycles and provide additional security. 

While many studies have focused on bicycling related to injury and pollution exposure [21,29,30] and other studies have focused on bicycle-related infrastructure and preferences [2,23,31,32,33], no studies have been conducted on existing wide cycle tracks in China to learn bicyclists’ perceptions of safety from having to share the wide cycle tracks with moving/parked cars and bus stops. Furthermore, no studies have explored bike parking options available in China. As college students comprise the largest share of bicyclists in China, this study investigated college students’ opinions about cycle tracks and on-campus parking in Beijing, China. 

This study could provide universal knowledge about cycle tracks because in cities, such as Montreal, bicyclists are stating that the existing cycle tracks are too narrow [34]. If cities widen their cycle tracks without careful design, vehicle drivers could enter. This study could also provide universal knowledge about bike parking because, unlike China, countries such as the U.S. provide inferior bike racks on the sidewalks, basement bike parking in car parking garages and bike parking cages on campuses in which students have to lift their bikes up to the second tiered bike rack if no space is available on the ground-level tier. Lessons learned from college students in China could provide insights about cycle tracks and bicycle parking that would be informative to urban planners and designers in China and other countries. 

## 2. Methods, Study Populations and Locations

There were three parts of this study: (1) cross-sectional survey questions that identified personal preferences about road types for bicycling and issues with cycle tracks; (2) cross-sectional survey questions that identified determinants of bicycle parking problems and solutions on a college campus; and (3) a scan of the bicycle parking environment on a college campus that presented different types of bicycle parking, capacity and vacancies at the school/office and living areas. The survey and scan were conducted from 17 to 19 May 2011 (UTC+8). This study applied the theory of transport policy using the Maslow Transportation Level of Service (LOS). For the cycle tracks and bike parking in China, only after the basic needs of safety from crash and security and fear of theft in bike parking are met can higher needs be met such as convenience and comfort [35].

The Maslow Transportation Level of Service (LOS) was applied because the current LOS focuses on automobiles. A road designated as LOS A for automobiles would mean drivers could maintain average speeds. This same road would be F for bicyclists because they would have to share the space with faster moving vehicles, a condition under which few would bicycle. By applying Maslow’s Transportation Level of Service, the needs for safety and security for all users must be met. On the same travel route, the designation of LOS A for vehicles should also mean an LOS A for bicyclists. If the vehicles are traveling at average speeds on a road designed for their safety and security, a barrier-protected cycle track should be provided to assure the safety and security of the bicyclists. The Maslow Transportation Level of Service was also applied to bike parking. In this case, after the lower needs of safety and security in bike parking are met, the higher needs of comfort and convenience can be met. If the travel routes for bicyclists meet at least the minimum needs of safety and security and bike parking is able to also affordably meet the higher needs of comfort and convenience, the numbers of bicyclists should increase. 

The cross-sectional in-dorm survey was distributed at Peking University Health Science Center (PKUHSC) in Beijing to undergraduate, masters, doctoral and junior college students who were living on campus. Random cluster (dormitory room) sampling was used to select participants, and verbal informed consent was collected from all who volunteered to participate. No identifying information was collected. The survey consisted of personal characteristics (age, gender, nationality, school year, school, etc.), personal bicycling habits (time spending riding per day and days spending riding per month, etc.), factors influencing bicycle use, perceived safety for four types of bicycle facility designs and three types of cycle track conditions, perceived problems with the bicycle parking environment on campus and suggestions to improve the bicycle parking environment. The survey questionnaire was pilot-tested and revised to reduce unclear wording. Among current students on the PKUHSC campus, 583 students (freshmen to graduate students) were drawn from 235 rooms (out of 1673) from six residence halls. Investigators distributed questionnaires to each room and then collected them after completion. In total, 425 students (61.9% female) from 209 rooms responded (73% response rate).

The PKUHSC campus includes 5 areas with 18 school/office buildings and 6 living areas with residential halls for students. The typical distance between the school/office buildings and living areas is about 1 km, a distance that can be covered in a 10-min walk or a 5-min bicycle ride. In total, there are 7 bicycle parking places in the school/office areas and 10 in living areas on campus.

The study met the criteria for exemption decided by the Institutional Review Board of Peking University Health Science Center. The Institutional Review Board of Harvard T.H. Chan School of Public Health further categorized the analysis as Not Human Subjects Research. Other investigators who are interested in accessing the dataset or replicating the analyses can contact the corresponding authors. All analysis was performed using Stata 12 (StataCorp, College Station, TX, USA).

### 2.1. Cross-Sectional Survey Questions about Road Types and Cycle Track Conditions

**Figure 1:**
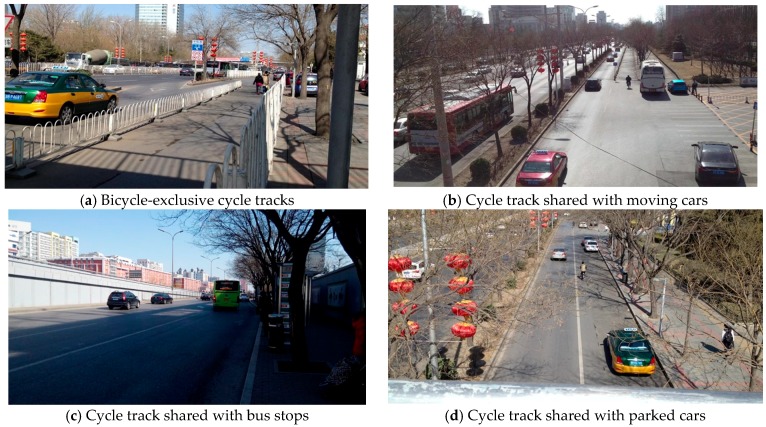
Design and cycle track sharing near the Peking University Health Science Center (PKUHSC) campus in China.

After excluding those who had missing values for preference or perceived safety, a total of 410 participants was included in the analysis. Perceptions of safety were compared among China’s four main bicycle route types: (1) mixed traffic (roads shared by car drivers, bicyclists and pedestrians); (2) roads shared by car drivers and bicyclists; (3) painted bike lanes between moving cars and the sidewalk; and (4) cycle tracks separated by a physical barrier. Perceived safety was also assessed on cycle tracks that have other users within the right-of-way, resulting in 3 encroachment conditions of the cycle tracks: a, parked cars; b, bus stops; and c, moving cars (Figure 1 shows four bicycle routes near PKUHSC campus in China, and Supplementary Table S1 shows the drawing-aided questions). For each road type or cycle track condition, perceived safety was categorized into five levels: very safe (1), safe, neutral, unsafe, to very unsafe (5).

The Kruskal –Wallis test was used to test the overall and pair-wise difference in perceived safety between different bicycle routes (cycle tracks as reference group) and between the different cycle track conditions (cycle tracks shared with bus stops as reference group). We adjusted for multiple comparisons using Bonferroni correction; therefore, we rejected the null hypothesis for *p*-values less than 0.05/6 = 0.009 for the four groups’ comparisons and 0.05/3 = 0.017 for the three groups’ comparisons. We also evaluated whether individuals’ perceptions of safety and preference were different across gender (female, male) and bicycle use status (yes, no).

### 2.2. Cross-Sectional Survey Questions about Bicycle Parking Problems and Solutions

Students were asked about problems with the bicycle parking environments on campus in school/office areas and in living areas. Questions centered on convenience, orderliness, security (guard or camera), types of racks and existence or lack of a shed. Questions were asked about how to improve the bicycle parking environment including perceptions about no need to improve, or improve the sheds, security, racks, other or security. Participants were also asked if they would bicycle more if parking were improved. Pearson’s chi-squared test was used to test the difference of perceptions by gender. All tests were two-sided with statistical significance set at *p* < 0.05.

### 2.3. Scan of the Campus Bicycle Parking Environments

**Figure 2:**
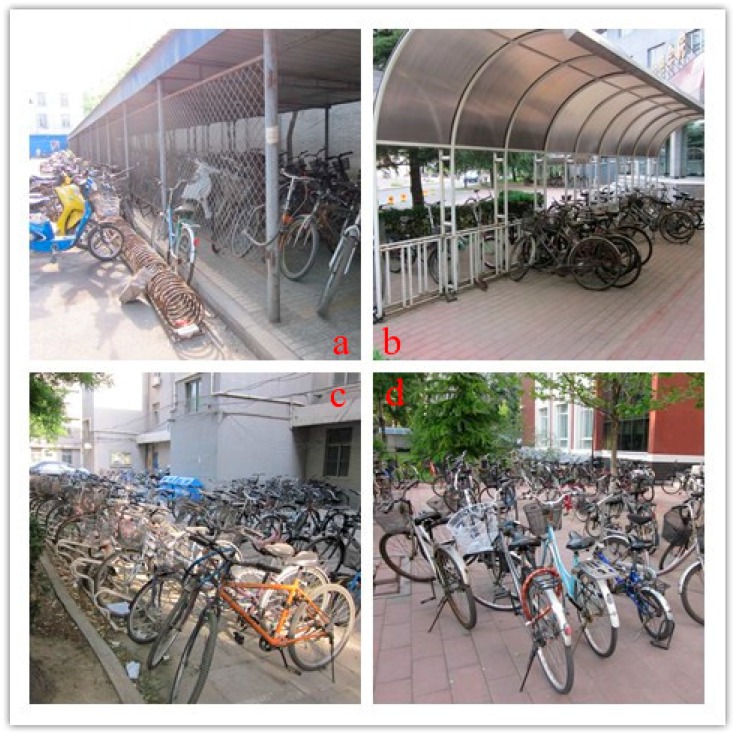
Four types of bicycle parking spots on campus. (**a**) With bicycle lockers/racks, a shed and bicycle services (pump and bicycle repair service). (**b**) With bicycle lockers/racks and a shed, but no bicycle services. (**c**) With bicycle lockers/racks, but no shed or bicycle services. (**d**) Without bicycle lockers/racks, a shed or bicycle services.

On this campus, one trained investigator conducted the scan on bicycle parking environments by observing the 17 bicycle parking places in the school/office areas and living areas on campus. A template was used to record the details of bicycle parking spots, including the numbers of parked motorcycles, parked bicycles, bicycle lockers, bicycle lockers in use, spaces of sheds in the parking area and spaces of sheds in use, as well as whether bicycle parking services and security (guard or camera) were provided. Four types of bicycle parking spots were catalogued on campus (Figure 2).

The counts were conducted on weekdays and weekends, but were not also conducted with factors of weather, time (morning, afternoon, and evening), summer vacations, etc. Weather cannot be changed, and thus, bike parking design would not be altered for weather except to provide a roof in geographic areas with high rainfall or roofs and walls in geographic areas with excessive snow. Time and vacations would be highly variable as some campuses might have many day students, while other campuses would serve individuals who work full time and attend night classes. Most campuses have marked differences between weekdays and weekends, and thus, those factors were included.

## 3. Results

Among the 410 college students in our analysis, almost half bicycled (49.8%), and 79.7% male and 56.9% female students were physically active (Table 1). For bicycle use, 24.7% of male students and 27.2% of female students bicycled 1–10 days per week, but, as the days increased to more than 20 days per month, fewer females bicycled this many days or more per month (3.9%) compared to males (23.4%). Of the surveyed students, most were from the schools of clinical medicine and basic medical science. For the analysis about parking preferences, after excluding those who had missing values for preference or perceived safety, a total of 381 participants were included.

### 3.1. Cross-Sectional Survey Results of Different Road Types and Cycle Track Conditions 

As indicated in Figure 1, most roads in China have 10.8–13 feet-wide cycle tracks; however, those cycle tracks are also shared with moving cars, parked cars and bus stops. Cycle tracks were perceived as safer compared with mixed traffic roads shared with cars and pedestrians, roads shared with cars or painted bike lanes (Table 2). Students ranked their perceived safety from most safe (one) to least safe (five) for the different facilities in the following order: cycle tracks separated by physical barriers (mean = 1.5); bicycle lanes with painted lines (mean = 2.4); roadways shared by car drivers and bicyclists (mean = 3.8); mixed traffic (shared by car drivers, bicyclists and pedestrians, mean = 4.4). An overall difference in perceived safety (*p* < 0.001) was observed among different routes. Among the four route types, exclusive cycle tracks type was deemed optimal in safety. Cycle tracks shared with moving cars, bus stops and parked cars were considered unsafe (*p* < 0.001). Amongst the three shared uses, sharing with moving cars was perceived as most unsafe (*p* < 0.001), while the perceived safety was not significantly different between the other two conditions (*p* = 0.30). Those who never bicycle and bicyclists equally perceived sharing with moving cars as unsafe (mean = 4.6) compared with sharing with a bus stop. Females perceived lower safety in sharing cycle tracks with moving cars (mean = 4.6) than males (mean = 4.5). Males and females showed less apprehension with cycle tracks that were shared with parked cars or bus stop users and more apprehension sharing with moving cars.

### 3.2. Cross-Sectional Survey Results about Bicycle Parking Problems and Solutions

Students identified lack of a shed at the bicycle parking area as a problem (55.8% male and 59.9% female), as well as at living areas (57.8% males and 56.0% females) (Table 3). Lack of security (guard or camera) was the second ranked problem at school/office areas (52.0% males and 56.4% females) and living areas (50.7% males and 44.1% females). Lack of order in the bicycle parking area was the third most highly listed problem in school/office areas (40.9% males and 32.6% females) and living areas (50.0% males and 43.6% females). When asked how to improve bicycle environments, students wanted more bicycle parking areas at school/office areas (80.5% males and 77.5% females) and at living areas (77.9% males and 75.8% females). The most suggested bicycle facilities were sheds, security (guard or camera), bicycle racks and bicycle parking services (pumps, etc.). If bicycle parking were improved, approximately three quarters of males and females indicated they would bicycle more. There were no statistically-significant differences between gender for the problems identified and suggestions except for “no need to improve”, with females indicating a greater desire for improvement in the living area. 

### 3.3. Scan of the Campus Bicycle Parking Environments

In the school/office areas, there were seven bicycle parking locations, and four of them were covered sheds, totaling 320 covered parking spaces (Table 4). At all of the bicycle parking spots in the school/office areas, there were no bicycle services (pump or bicycle repairs), and there was no security (guard or camera). For racks during the weekday, only 20.8% were in use, and on weekends, 10.0% were in use, while for sheds during the weekday, 48.4% of the shed spaces were in use and on weekends 39.4%.

In the living areas, there were 10 bicycle parking locations, and two of them were covered sheds. In the living areas, one spot had bicycle services (pump or bicycle repairs) and none had security (guard or camera). Compared to school/office areas, the occupancy rate of shed spaces at living areas was higher at 71.1% on weekdays and even higher at 74.4% on weekends. On the weekend in the living areas, a total of 1597 bikes were parked. With 770 spots available inside the caged shed and only 573 of those covered parking spaces occupied, the students did not fill all of the parking spaces inside the caged parking sheds.

Besides the school/office building, complexes and living areas are large open paved areas where bicycles can be parked using kickstands. These areas are less accessible to the general public, thus deterring theft. These areas also contain bicycle racks and covered bicycle parking sheds, some with chain link fencing on the walls and others with open sides and only a roof. These areas are close to the school/office space or the living space, thus not requiring significant time walking before or after parking. Even in crowded China, all campus bicycle parking provisions involved leaving the bicycles horizontal and on the ground. The bicycles were not parked in a second tiered rack above other bicycles or hung on a wall.

## 4. Discussion

In this study on bicycle environments in China, half of the Peking University students surveyed bicycle, and though they prefer cycle tracks more than the other bicycle facilities, China’s wide cycle tracks now also have moving cars, parked cars and bus stops. The most disliked encroachment was the moving car, perhaps because moving cars operate at a high speed and can be driven unpredictably in multiple directions while a parked car or a bus/passenger poses a lower risk due to predictability. Close to half thought campus bike parking lacked order. The most suggested bicycle parking facilities were sheds, security (guard or camera), bicycle racks and bicycle parking services (pumps, etc.). If parking were improved, three quarters indicated they would bicycle more. Caged sheds were preferred but, in living areas with 1597 parked bikes, caged sheds were only 74.4% occupied. 

### 4.1. Perceptions of Safety of Bicycle Environments

Although bicycling is a personal behavior, route design plays an important role in an individual’s decision to bicycle [3,9,10]. Similar to the perception by the students that cycle tracks were safest, studies in other countries have suggested preference for bicycle facilities separated from vehicular traffic [36]. In a study in Montreal, 2.5-times as many bicyclists chose the cycle track compared to a comparable road without bicycle provisions [12]. Factors that discourage or encourage biking include having to bicycle with mixed traffic, the presence of a cycle track and the existence of on-street parking [8,37]. Studies have also shown preference for routes with traffic calming, cycle tracks, paved surfaces and no on-street parking [2,8]. In a preference experiment of 1128 current cyclists in Canada, bicycling in mixed traffic (i.e., no facilities) was considered the worst scenario [2]. 

Individuals have also been exploring how to make bicycling safer [22,38]. A study in Montreal found that cycle tracks had a 28% lower injury rate, and another study in Canada showed that a cyclist was ten-times as likely to be injured on a busy street with parked cars than on a cycle track alongside the street that is separated by a physical barrier [12,15]. In this study, female students, more than male students, perceived cycle tracks shared with moving vehicles as unsafe. The gender difference is consistent with research conducted about the cycle tracks in Hangzhou, China, where the cycle tracks are wide and bicycle-exclusive, that found females rated bicycling with vehicular traffic less safe than men [18].

Our study differs from these previous studies because those cycle tracks were bicycle-exclusive, including the cycle tracks in Hangzhou, China. The cycle track transportation networks in China have been in place since 1949 [14,39]. With the introduction of more vehicles, the prestige of owning and driving a car and the crowding of Chinese cities, the wide cycle tracks in China are now also used for moving vehicles, parking cars and waiting for buses. The students in our study disliked having to share the cycle tracks with moving vehicles more than their dislike of sharing with parked cars or bus stops. Many studies in Western countries revealed the hazard of serious crashes from on-street car parking due to visual obstruction and being injured by opening car doors [40,41]. Even if the door of a parallel-parked car opens on a cycle track, there is less likelihood that a moving vehicle will simultaneously be passing by on the cycle track.

### 4.2. Informing the Future Design of Cycle Tracks

For future cycle tracks, our findings suggest that on the existing wide cycle tracks in Beijing, the students least preferred sharing with moving cars. For a car to be parked, it has to move to get to the parking space. Drivers of cars can drive in unpredictable directions and at a high speed, raising the apprehension of bicyclists. Therefore, the wide cycle tracks should not be shared with moving or parked cars. Sharing with bus stop users was not preferred, but in crowded Chinese cities, perhaps the wide cycle tracks have to be shared. Therefore, one consideration could be putting a lane for Bus Rapid Transit (BRT) [42] on half of the cycle track. Metal fences could separate the bus from the bicyclists, a common divider used on Chinese cycle tracks [14]. BRT would have few bus stops, so there would not be many bus stops in which passengers would cross the cycle tracks. Alternatively, the wide cycle track could be shared with a local bus, again with the metal fence as a division. Where passengers have to get on and off, a raised island could be provided for bus passengers to wait for the bus (Supplementary Figure S1). As the number of bicyclists increase in China riding their own bikes, using shared bikes and riding E-bikes, the new policy could alternatively be to ban cars and buses on the wide cycle tracks. 

In the U.S. and other countries where the cycle tracks are less wide (e.g. one way/five feet minimum width in the U.S. and two-way/9.8 feet wide in Montreal), moving cars, parked cars and bus stops might appear less critical. Yet, as the numbers of bicyclists increase, cycle tracks will become wider to allow for side-by-side riding, bicyclists to pass and child-carrying cargo bikes. New cycle track designs should deter entrance by drivers. If bus stops are near the cycle tracks, the buses should have a bicycle-separate corridor for approaching the bus stop, and the bus passengers should have a cycle track-separate space for waiting. 

### 4.3. Bicycle Parking Opinions

The most often identified problems with bicycle parking were: (1) lack of a shed; (2) no security (guard or camera); and (3) bicycle parking was not orderly. Moreover, insufficient bike parking space and concerns over bike theft were major reasons for the underuse of bicycles. If bicycle parking conditions were improved, three quarters of the respondents indicated that they would increase their bicycling. These results are consistent with a study of bicycle parking in the central business district of Shanghai, China, where they found that bicycle parking was greatly oversaturated and parking management was insufficient [43]. Another study from Shanghai found that the major reasons for not using the bicycle were inconvenience, inadequate bike parking facilities and fear of bicycle theft, which are similar to our study [44]. Studies both in Australia and the U.S. also found that the provision of secure bicycle lockers on campus would foster cycling among college students [45,46].

For the short distance from their campus living area to the office/school area, the students would have to remove their bicycle from the living area parking. The scan revealed that parking sheds in the living areas, which were the most preferred facility, were about three quarters full. To remove a bicycle from the shed to bicycle the short distance to the offices/school and park their bicycle again might not have been worth the effort or the risk of having the bike stolen at the office/school. The returning student would also then have to find a parking slot in one of the two sheds in the living areas. 

### 4.4. Informing the Future Design of Bike Parking

While the optimal occupancy rate for car parking garages has been identified as 85–95% [47], the best occupancy rate for bike parking has not been identified [48,49]. Designers of car parking garages do not aim for 100% occupancy because they want to deter drivers from moving from floor to floor. Unlike with cars that stay separated and have designated parking spaces, bikes can come in contact with other bike’s handlebars, pedals and gear, causing bike-on-bike damage. The Dutch Board of the Stichting FietsParKeur recommends a minimum of no more than five maneuvers (shifting, pushing, pulling or tilting) when parking a bike [50]. Having a bicycle parking shed at a 74% occupancy rate might be considered too full, and thus, bicyclists do not prefer to use the caged shed. To enable more bicyclists to benefit from the weather protection, caged sheds should be designed to achieve 100% occupancy with sufficient space to deter bike damage and with few maneuvers.

For the bike parking, the most desired facility was a shed with a guard and with order. Bikes in the campus sheds are parked on the ground and not hung on the wall or put in a top tier rack. Though wall mounts and tiered parking are more orderly plus space-saving, and thus encouraged in the U.S., hanging or lifting a bike is more difficult for a female if the bike is heavy. As it would be expensive to have a guard at each shed, perhaps a state-of-the art bike parking shed could be designed with not just racks for slipping in a bike (and having bike-on-bike damage), but combining the preference of the large parking area (Figure 2d) with car parking painted spaces. Parking on the campus was deemed disorderly and perhaps, similar to white lines for car parking, bikes could also have a designated space. Each bike space could have one single upside down U in the painted bike parking space.

Bicycles on the school/office areas were indicated as not being parked in an orderly manner. There was also an indication that shed parking was lacking, but only 48.4% of spaces in the school/office sheds were taken. The occupancy rate of sheds was much lower than expected, which is similar to a recent study in Hangzhou where the use of bicycle parking sheds (39.8%) was much lower than the preference of parking sheds by all study participants [18]. This suggests that interventions could include telling students about the sheds, instructing them to park in a more orderly manner in the sheds and also better designing of the shed parking, so leaving a bike involves few movements, order is automatic and bikes are not scratched.

## 5. Strengths and Limitations

There are several strengths in our study. To our knowledge, this is the first opinion survey on bicycle route preference and perceived safety in Beijing, a highly crowded city. Visual aids with drawing descriptions were used to better define the bicycle environments. We also included a range of bicycle environments in addition to the traditional broad categories of cycle tracks/streets/paths to ensure common and correct understanding of terminology [2,51]. No other country has wide cycle tracks like China, and the lessons from Beijing could be used to inform future cycle track designs if wide cycle tracks are built. In addition, the findings about bicycle parking on school/office and living areas could provide insights about issues and problems with existing bicycle parking environment and inform future design and improvement of the bicycle facility, which could ultimately promote bicycle use and physical activity among the public. The study provides a framework for others who may wish to conduct similar research. 

However, the study has limitations. The response rate was 73%, and the non-response, although modest, may have reduced the sample representativeness. The self-reported approach made our study prone to potential under-/over-estimation of individuals’ health behaviors. Secondly, the questionnaire used in this survey was self-designed by the authors and geared for bicycle use, route preference and environmental conditions on a campus in China. The findings might not reflect all of the factors worldwide about bicycle parking, but the choice of “other” was offered on the questionnaire. Therefore, participants had the opportunity to introduce a wider range of bicycling or bicycle parking issues. Last, only four basic bicycle environments were included, and more detailed and important characteristics including street illumination, hills and intersections could be considered. Thus, further detailed research is warranted. 

## 6. Conclusions

Our study addresses the negatives about having wider cycle tracks before these negatives become a problem. The recent bike share boom in China indicates that need for well-maintained and further-refined bicycle environments [52,53]. As cities across the world build more cycle tracks, they should identify effective ways to deter cars from driving and parking on the cycle tracks. If a country knows in advance that these could become issues and they need to widen their cycle tracks, they can design the cycle tracks differently (Supplementary Figure S1: wide bus stop islands on the street side of the road, etc.) and also develop policies (give a ticket to a driver parking or driving in the cycle track). Given participants’ perceived safety of the cycle tracks, especially after eliminating moving/parked vehicles and buses, cycle tracks should continue to be built and maintained as bicycle-exclusive networks in China and elsewhere. If transit is necessary and the cycle tracks are wide, Bus Rapid Transit (BRT) or local buses could be on one side in a wide cycle track with a divider between the buses and the bicyclists. If other countries widen their cycle tracks, vehicles should be deterred from entering.

Moreover, bicycle parking can be improved, a change that is modifiable, unlike the weather or distance. Improving bicycle parking is achievable by an institution, and not as costly as the governmentally-regulated task of improving bicycle routes. Bicycle parking improvements on a campus could include providing parking sheds, services at parking areas (pump and repairs), security (camera, guard or the bicycle repair person’s surveillance), orderliness through paint and adjusted bike parking occupancy percentages to lessen bike-on-bike damage and keep to a minimum the number of times a bike has to be manipulated to be parked. Bike parking sheds could be redesigned with painted lines to offer more space and order, similar to car parking. 

By applying Maslow’s Transportation Level of Service (LOS) to the travel routes, LOS A for the vehicles and LOS A for the bicyclists better guarantees meeting, at a minimum, the basic needs of safety and security for both vehicle occupants and bicyclists. If Maslow’s Transportation LOS is applied also to bike parking, safety and security needs can be met, but the higher needs of comfort and convenience could also be met. With safer routes and enhanced bike parking, the numbers of bicyclist could increase. Overall, as a crucial determinant of bicycle behavior and sustainable transportation, physical environments for bicycling that are preferred and considered safe can be created and improved to benefit the general public [54,55].

## Figures and Tables

**Table 1:** Overall and gender-specific characteristics among participants.

Variable	Overall (N = 410)		Male (N = 155)		Female (N = 255)	
	n	%	n	%	n	%
Age (N = 408)						
18–23	239	58.6	88	57.5	151	59.2
≥24	169	41.4	65	42.5	104	40.8
Physical activity (N = 394)						
Met WHO requirement	258	64.8	118	79.7	140	56.9
Physical inactivity *	136	35.2	30	20.3	106	43.1
Bicycle use (days/month) (N = 408)						
1–10 days	107	26.2	38	24.7	69	27.2
11–20 days	50	12.3	18	11.7	32	12.6
21–30 or 31 days	46	11.3	36	23.4	10	3.9
Never	205	50.3	62	40.3	143	56.3
School (N = 409)						
Public health	59	14.4	11	7.1	48	18.8
Clinical medicine	86	21.0	38	24.7	48	18.8
Dental medicine	28	6.9	16	10.4	12	4.7
Basic medicine science	100	24.5	38	24.7	62	24.3
Foundational education	16	3.9	9	5.8	7	2.8
Nursing	62	15.2	8	5.2	54	21.2
Pharmaceutical science	58	14.2	34	22.1	24	9.4

* Physical inactivity: less than 150 min of moderate-intensity physical activity, or less than 75 min of vigorous-intensity aerobic physical activity, or an equivalent combination of moderate- and vigorous-intensity activity throughout the week.

**Table 2:** Perceived safety of road type, cycle track conditions, cycle track sharing-comparison overall, bicycle use and gender.

Variable	Perceived Safety *		
	Median	Mean	*p*-Value ^ξ^
**Road Type**			
Mixed traffic	5	4.4	<0.001
Roads shared with cars	4	3.8	<0.001
Painted bicycle lanes	2	2.4	<0.001
Cycle tracks	1	1.5	ref
**Cycle Track Condition**			
Exclusive cycle tracks	1	1.5	ref
Shared with parked cars	4	4.1	<0.001
Shared with bus stop users	4	4.1	<0.001
Shared with moving cars	5	4.6	<0.001
**Cycle Track Sharing**			
**Overall (N = 420)**			
Shared with parked cars	4	4.1	0.30
Shared with bus stop users	4	4.1	ref
Shared with moving cars	5	4.6	<0.001
**Bicycle users (N = 230)**			
Shared with parked cars	4	4.1	0.21
Shared with bus stop users	4	4.1	ref
Shared with moving cars	5	4.6	<0.001
**Never Bicycle (N = 205)**			
Shared with parked cars	4	4.1	0.43
Shared with bus stop users	4	4.2	ref
Shared with moving cars	5	4.6	<0.001
**Male (N = 155)**			
Shared with parked cars	4	4.1	0.04
Shared with bus stop users	4	3.9	ref
Shared with moving cars	5	4.5	<0.001
**Female (N = 255)**			
Shared with parked cars	4	4.2	0.24
Shared with bus stop users	4	4.2	ref
Shared with moving cars	5	4.6	<0.001

* Higher score indicates less safe. ^ξ^ Kruskal–Wallis test was used for pairwise comparisons and *p*-value was only given when comparing to the reference group.

**Table 3:** Problems with and suggestions on how to improve bicycle parking environment on campus *. (N = 381).

Problems	School/Office Areas			Living Area		
	Male	Female	*p*-Values ^ξ^	Male	Female	*p*-Values ^ξ^
N	154	227		154	227	
No problem (%)	3.9	9.7	0.054	3.3	8.4	0.074
Problems (%)						
Lack space for bicycle parking area	29.2	26.9	0.63	33.1	30.0	0.53
Bicycle not parking orderly	40.9	32.6	0.098	50.0	43.6	0.21
Lack shed at bicycle parking area	55.8	59.9	0.45	57.8	56.0	0.72
Lack bicycle racks at bicycle parking area	21.4	27.3	0.24	27.9	25.6	0.61
Existing racks are inconvenient	14.3	9.3	0.13	16.2	12.3	0.27
Bicycle parking has no security (guard or camera)	52.0	56.4	0.46	50.7	44.1	0.25
Lack of convenient bicycle services nearby	18.2	26.4	0.056	18.2	18.5	0.94
Others	1.3	2.2	0.52	0.65	2.2	0.23
Suggestions (%)						
No need to improve	10.4	14.1	0.37	8.4	16.3	**0.035**
Provide parking area for bicycle parking	80.5	77.5	0.52	77.9	75.8	0.64
Sheds	46.1	50.2	0.44	45.5	46.3	0.88
Security (guard or cameras)	40.3	37.0	0.56	39.0	32.6	0.22
Bicycle racks	33.8	33.9	0.98	35.1	32.2	0.57
Others	0.65	0.0	0.22	0.0	0.44	0.41
Provide bicycle parking services	27.3	36.1	0.098	26.6	30.8	0.43
Others	0.7	1.3	0.53	2.0	0.0	0.033
I will probably increase bicycle use if bicycle parking environment is improved (%)	71.4	70.5	0.86	74.7	68.3	0.20

* Excluded surveys with missing values for preference or perceived safety. **^ξ^** *p*-value from chi-square test, 2-sided; bold values indicate *p* < 0.05.

**Table 4:** Number of bicycle parking spots scanning on campus.

Type of Parking Spot	School/Office Areas		Living Area	
	Weekdays	Weekends	Weekdays	Weekends
Parking location, N	7	7	10	10
With sheds, N	4	4	2	2
With bicycle services, N	0	0	1	1
With security, N	0	0	0	0
Parked motorcycles, N	38	10	30	17
Parked bicycles, N	511	394	1590	1597
Bicycle lockers, N	120	120	816	816
Bicycle lockers in use, N (%)	25 (20.8%)	12 (10.0%)	148 (18.1%)	162 (19.9%)
Spaces of sheds on parking area, N	320	320	770	770
Spaces of sheds in use, N (%)	155 (48.4%)	126 (39.4%)	547 (71.0%)	573 (74.4%)

## Supplementary Materials

The following are available online at www.mdpi.com/1660-4601/14/8/930/s1, Figure S1: Cycle track and bus stop design in Australia, Table S1: Picture-aided bicycle route questions in the survey.
