# Supplementary material for: Cycle Tracks and Parking Environments in China: Learning from College Students at Peking University
Source: Int J Environ Res Public Health. 2017 Aug 18;14(8):930. doi: 10.3390/ijerph14080930 (PMC5580632; doi:10.3390/ijerph14080930)
Supplement: Supplementary file 1 [file ijerph-14-00930-s001.pdf]

International Journal of
Environmental Research and Public Health

MDPI

Supplementary Materials: Cycle Tracks and Parking Environments in China: Learning from College Students at Peking University

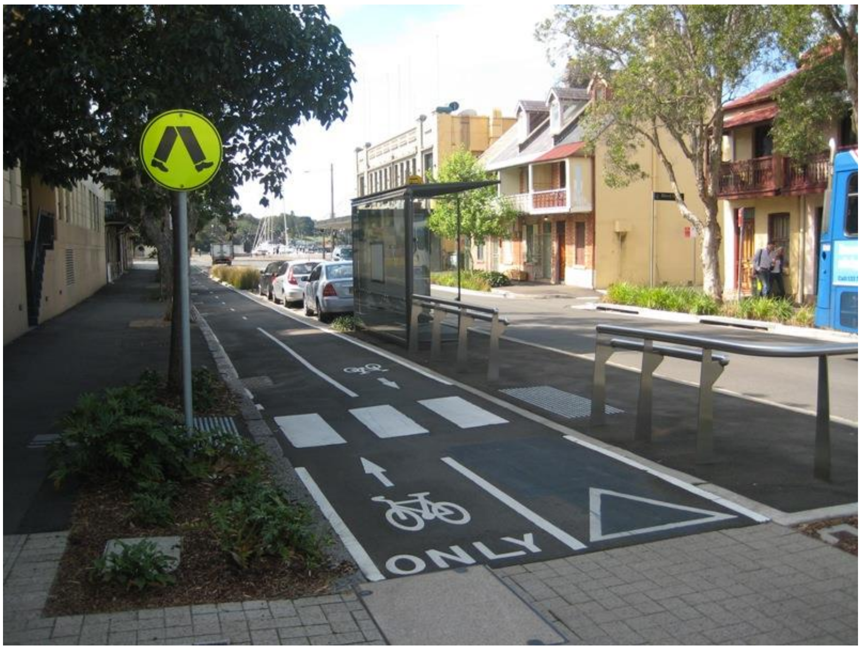

Figure S1. Cycle track and bus stop design in Australia.

Table S1. Picture-aided bicycle route questions in the survey.

Route Type/Condition	Picture	Preference *	Perceived Safety ξ
Route 1: Mixed road for cars, bicyclists and pedestrians	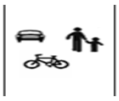	①–⑤	①–⑤
Route 2: Mixed road for cars and bicyclists	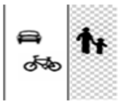	①–⑤	①–⑤
Route 3: Bicycle lanes separated by painted lines	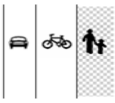	①–⑤	①–⑤
Route 4: Cycle tracks (barriers separating cycle tracks from moving cars include metal barrier, trees and other plants, etc.)	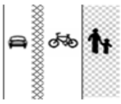	①–⑤	①–⑤
Condition 1: Cycle track sharing with parked cars	N/A	①–⑤	①–⑤
Condition 2: Cycle track sharing with bus stops	N/A	①–⑤	①–⑤
Condition 3: Cycle track sharing with moving cars	N/A	①–⑤	①–⑤

* ① Strongly preferred, ② Preferred, ③ Neutral, ④ Not preferred, ⑤ Strongly not preferred.
ξ ① Very safe, ② Safe, ③ Neutral, ④ Unsafe, ⑤ Very unsafe.
